# Implication of single year seasonal sampling to genetic diversity fluctuation that coordinates with oceanographic dynamics in torpedo scads near Taiwan

**DOI:** 10.1038/s41598-020-74025-9

**Published:** 2020-10-08

**Authors:** Yong-Chao Su, Shan-Hui Su, Han-Yun Li, Hurng-Yi Wang, Sin-Che Lee

**Affiliations:** 1 Department of Biomedical Science and Environmental Biology, Kaohsiung Medical University, Kaohsiung, 80708 Taiwan; 2 Kaohsiung Municipal Zhongshan Elementary School, Kaohsiung, 80457 Taiwan; 3 Institute of Clinical Medicine, National Taiwan University, Taipei, 10617 Taiwan; 4 Institute of Cellular and Organismic Biology, Academia Sinica, Taipei, 11529 Taiwan

**Keywords:** Ecology, Evolution, Zoology

## Abstract

Many fisheries management and conservation plans are based on the genetic structure of organisms in pelagic ecosystems; however, these structures tend to vary over time, particularly in cyclic ocean currents. We performed genetic analyses on the populations of the pelagic fish, *Megalaspis cordyla* (Osteichthyes: Carangidae) in the area surrounding Taiwan during 2000–2001. Genotyping was performed on *M. cordyla* collected seasonally around Taiwan as well as specimens collected from Singapore (Malacca strait) and Indonesia (Banda Sea). Gonadosomatic indices (GSI) revealed that *M. cordyla* does not spawn near Taiwan. Data related to the mitochondrial control region revealed that the samples from Singapore and Indonesia represented two distinct genetic cohorts. Genotyping revealed that during the summer (June–August 2000), the Indonesian variant was dominant in eastern Taiwan (presumably following the Kuroshio Current) and in the Penghu region (following the Kuroshio Branch Current). During the same period, the Singapore genotype was dominant along the western coast of Taiwan (presumably following the South China Sea Current); however, the number dropped during the winter (December–February 2001) under the effects of the China Coast Current. Divergence time estimates indicate that the two genetic cohorts split during the last glacial maximum. Despite the fact that these results are based on sampling from a single year, they demonstrate the importance of seasonal sampling in unravelling the genetic diversity in pelagic ecosystems.

## Introduction

Management plans for fisheries and the conservation of biodiversity in pelagic ecosystems are commonly based on geospatial data related to a target species^1–3^. The fact that the genetic structures observed in many pelagic fish are associated with oceanographic dynamics^4,5^ means that the conclusions drawn from genetic diversity and heterogeneity among populations are often attributed to connectivity among populations. This approach is well suited to deriving detailed descriptions of spatial genetic structures and identifying sources of genetic variation; however, the oceanographic dynamics associated with pelagic fish populations tend to be cyclic (i.e., changing seasonally or annually)^6–8^. It is crucial therefore to base plans for the conservation of pelagic fish stocks on the dynamics of genetic structures within the context of oceanographic cycles. Unfortunately, many studies on genetic differentiation among populations of highly migratory marine organisms rely heavily on one-time sampling at each sampling site. As a result, the cyclic dynamics of genetic diversity are often obscured. In this study, we sampled the pelagic fish stocks of torpedo scad, *Megalaspis cordyla* (Linnaeus, 1758) (Perciform: Carangidae) in the offshore ocean currents surrounding Taiwan. Note that sampling was performed at various time-points throughout the year to enable the analysis of seasonal variations in genotype diversity. Despite the fact that these results are based on sampling from a single year, our findings clearly illustrate the importance of using serial sampling to identify changes in cryptic genetic structures associated with oceanographic dynamics as well as the important implications this can have for conservation.

The torpedo scad (*M. cordyla*) is an important commercial species characterized by large scutes along the lateral line and a large black spot on opercle. This species is broadly distributed throughout equatorial areas of the West Pacific and Western Indian Oceans^9^. As a migratory pelagic fish, the torpedo scad is frequently found in areas ranging from southern Japan to the Indo-west-Pacific including Australia^10^. Trawling for *M. cordyla* in offshore regions around Taiwan peaks between April and November^11^. The strong swimming ability of this schooling species allows it to feed primarily on other fish. The body size is generally in the range of 30–40 cm, with exceptionally large specimens reaching 80 cm in total length (TL) and 3–4 kg in weight.

A case study conducted in south India (Vizhinjam)^9^ reported that individual fish reach sexual maturity in the first year, after growing to roughly 25 cm TL. They reported that prolonged spawning takes place once a year between December and July. They also reported that 90% of the total annual catch can be attributed to peak spawning during the previous monsoon season. The scad *M. cordyla* is far less important to fishing off the coast of China and other areas of East Asia, where the number of landings is ranked as minor. Larvae of the torpedo scad have been observed in abundance 5 km offshore in the region of southern India^12^; however, they are rarely observed off the coast of Taiwan^11^, indicating that Taiwan is not a major spawning area of this species. Most of the well-grown but immature torpedo scad appearing seasonally in areas near Taiwan come from equatorial areas via two main ocean currents.

The main ocean currents around Taiwan are the Kuroshio Current (KC), the South China Sea Current (SCSC), and the China Coastal Current (CCC)^13^. The KC is a northward deflection of the horizontal North Equatorial Current (near 13°N, Samar and Leyte, Philippines). The KC is a strong ocean current that runs constantly northward along the east of Taiwan to Ryukyu, reaching ~ 30°N off the coast of Japan^14,15^. The KC carries warm water (> 25 °C) from equatorial regions. In the Strait of Luzon, it branches toward the west of Taiwan, but this Kuroshio Branch Current (KBC) weakens near Penghu between April and November, such that none of the warm water extends north of Penghu between December and March^16^. The SCSC passes through the Bashi Channel and South China Sea moving north into the Taiwan Strait in the summer, whereas the CCC moves toward the south during winter^13,16^. The migratory *M. cordyla* originates in tropical regions; therefore, we predicted that the KC carries the Indonesian sourced individuals toward Taiwan via the Philippine Sea, whereas the SCSC carries the Indian Ocean sourced individuals via the South China Sea (only during the summer). No previous research has indicated whether there is any mixing of the discrete populations of *M. cordyla*. Furthermore, the time-course dynamics of the genetic structure of this species has yet to be elucidated.

Most of the mtDNA polymorphisms in pelagic migratory fishes present low intraspecific genetic divergence due to strong gene flow among populations^17–22^. A high degree of homogeneity has also been observed in the genetic makeup of other widely distributed oceanic organisms (e.g., sea urchins^23^, giant clams^24,25^, and starfish^26^), which drift along ocean currents during the larval stage. Some of the pelagic migratory species with life histories similar to that of *M. cordyla* present discrete genetic structures, despite the fact that they are widely dispersed throughout the West Pacific and East Asian oceans. There is considerable genetic heterogeneity among populations of scad mackerel (*Decapterus macarellus*) from the Molucca Sea and Banda Sea^27^. Populations of grey mullet (*Mugil cephalus*) in the East China Sea present distinct differences at two loci, GPI 100 and GPI 135^28^. Other nonmigratory but widely distributed fishes, such as damselfish^29^ (*Dascyllus carneus* and *D. aruanus*), also present genetic differences according to whether they appear in the Indian Ocean or the West Pacific Ocean. There are two general hypotheses pertaining to the evolutionary processes underlying the genetic differentiation of highly migratory marine organisms. The first hypothesis posits that discrete populations are isolated by existing oceanographic processes (e.g., ocean currents), and thus became non-panmictic. The second hypothesis attributes genetic differentiation to historical events (e.g., glaciation-related land barriers) resulting in genetically differentiated cohorts that are unable to interbreed^27,29–31^. Note however that many studies on migratory marine species rely on one-time sampling per site; i.e., they fail to consider the influence of oceanographic cycles on population divergence. The *M. cordyla* in the Indo-Pacific oceans and the amalgamation of the cohorts from different source populations in the offshore waters around Taiwan provide a rare opportunity to test the hypothesis that seasonal dynamics in the genetic structures of highly migratory pelagic organisms are associated with cyclic ocean currents. Our objectives in the current study were as follows: (a) to examine seasonal variations in the reproductive status of torpedo scad in the offshore waters of Taiwan; (b) to test whether different ocean currents on the eastern and western sides of Taiwan carry different genetic populations of torpedo scad; (c) to examine the correlation between seasonal ocean current dynamics and seasonal changes in the genetic structure of torpedo scad populations; (d) to estimate the time of population divergence and demographic changes in populations. Our results clearly demonstrate the importance of performing sampling at multiple timepoints in order to capture time-sensitive fluctuations in the genetic structure of migratory pelagic organisms and thereby elucidate the corresponding cryptic genetic diversity.

## Results

### Seasonal fluctuations in the gonadosomatic index (GSI)

**Figure 1:**
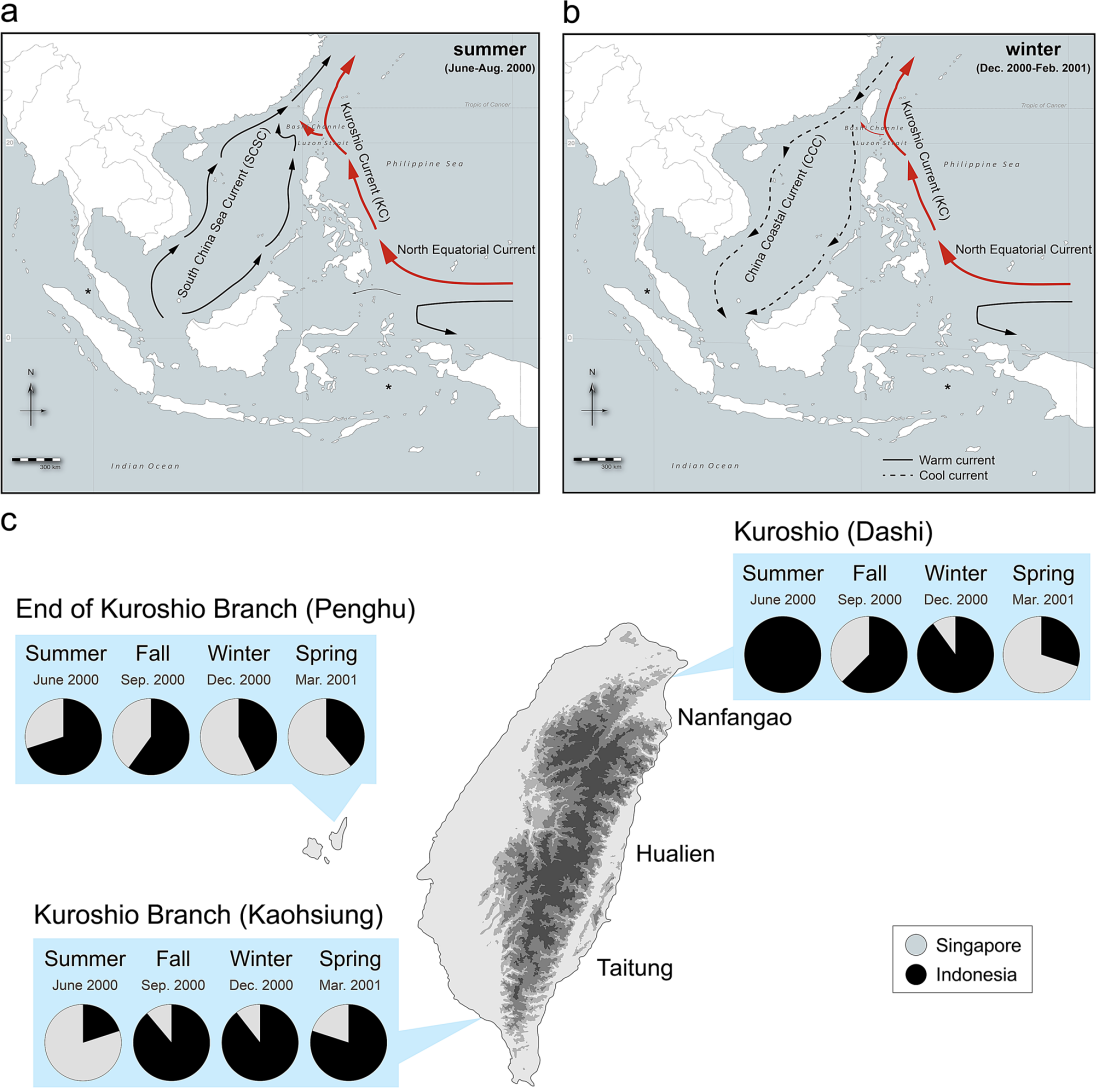
The cyclic oceanographic dynamics of the offshores near Taiwan based on the − 20 m Shipboard Acoustic Doppler Current Profiler (SADCP)^38^ data from June 2000 to May 2001. Collecting sites of Singapore and Indonesia samples are marked as asterisks. Geographical data based on the GADM database of Global Administrative Areas 3.6^46^. Map created using the Free and Open Source QGIS 3.12.3^47^ and was modified with Adobe Illustrator CC 2019^48^. (**a**) During the summer, both the warm currents, Kuroshio Current (KC) and Kuroshio Branch Current (KBC), showing in bold red lines, and South China Sea Current (SCSC), showing in bold black lines, are running to the north. (**b**) During winter and spring, while the KC still running toward north and KBC is weakening, the cold China Coastal Current (CCC) is running to the south, showing in dashed back lines. (**c**) The proportional fluctuations of the Singapore genotypes (gray) and Indonesia genotypes (black). The Indonesia genotypes are dominant in summer when KC is strong. On the west, the samples from Kaohsiung showed mixed genotypes with mostly Indonesia genotypes except for the summer. In summer, the Singapore genotypes are dominant, while SCSC is strong. The samples from Penghu, where at the end of KBC, the genotypes are mixed throughout a year. Summarized by Hu et al. (2000), Jan et al. (2002), Gallagher et al. (2009), and Gallagher et al. (2015)^13–16^.

**Figure 2:**
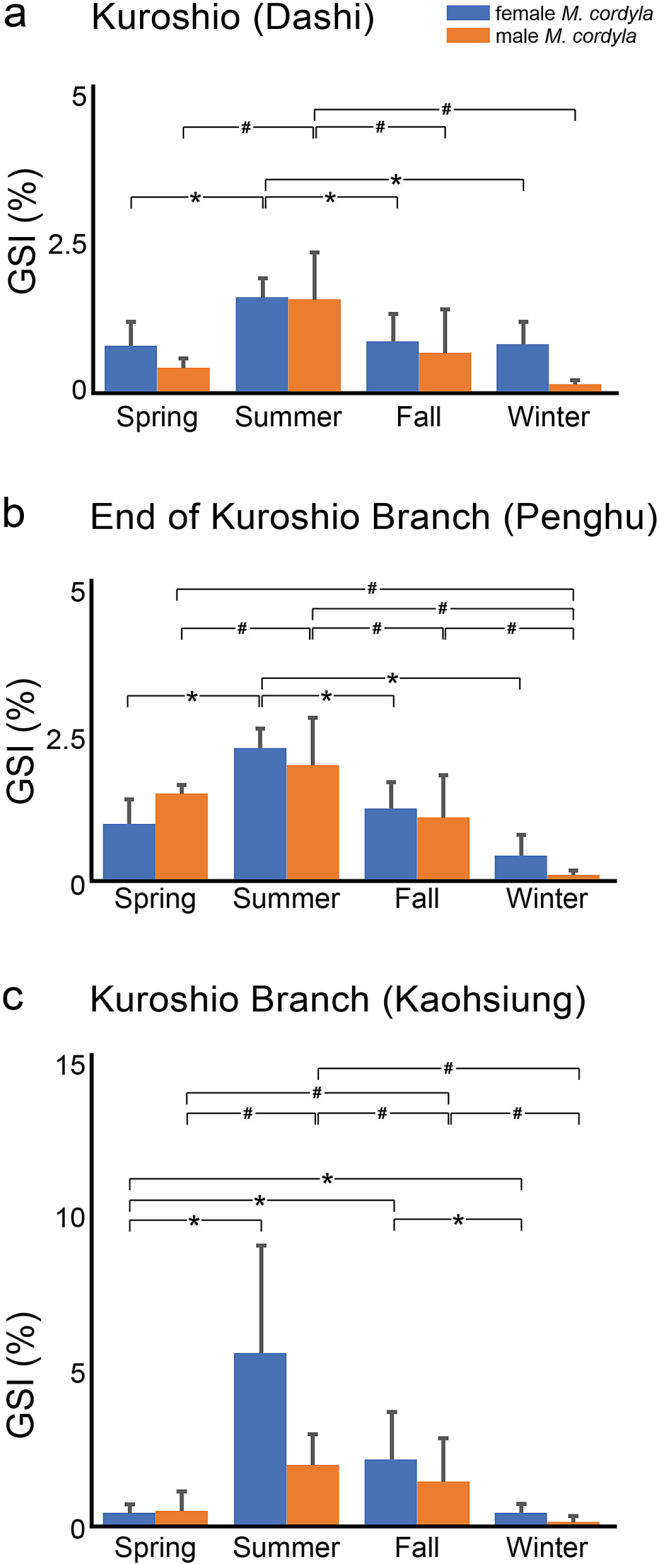
The ANOVA results of the gonadosomatic index (GSI) collected from the female (blue bars)/male (orange bars) samples of (**a**) Dashi, (**b**) Penghu, and (**c**) Kaohsiung. The significant difference among four seasons in the female and male is shown by asterisks as well as hashtags, respectively (*p* < 0.05). The samples collected from all seasons in three localities all showed that the GSI values are significantly higher in summer comparing to spring and winter. However, none of the GSI values reaches sexual maturity.

The seasonal changes in the GSI values of samples collected from Dashi (0.75–1.55%), Penghu (0.41–2.20%), and Kaohsiung (0.39–5.41%) (Fig. 1) were insufficient to indicate gonad maturity (> 5%; stage VI; ready for spawning) based on the criterion set for populations in the Indian Ocean^32^. The results of ANOVA and Duncan’s multiple range test revealed that the GSI peaked in the summer on the east coast (KC) as well as the west coast (KBC) of Taiwan, and was significantly higher at this time than during other seasons (*p* < 0.05) (Fig. 2; Supplementary Table S1). The body length of fish captured in Taiwan varied seasonally without notable fluctuations. The mean body length ranged from 24 to 35 cm, which exceeded the 25 cm corresponding to the size at maturity reported for populations in the Indian ocean^12^. Among the fish collected from Kaohsiung and Dashi, the body length of those sampled in the winter were longer than those sampled in the summer (*ANOVA*, *p* < 0.05) (Supplementary Table S1). These results are inclined to indicate that the main spawning grounds are not located in the waters near Taiwan, but rather in the tropical oceans to which they would eventually return^12^.

### Seasonal changes in genetic diversity

**Figure 3:**
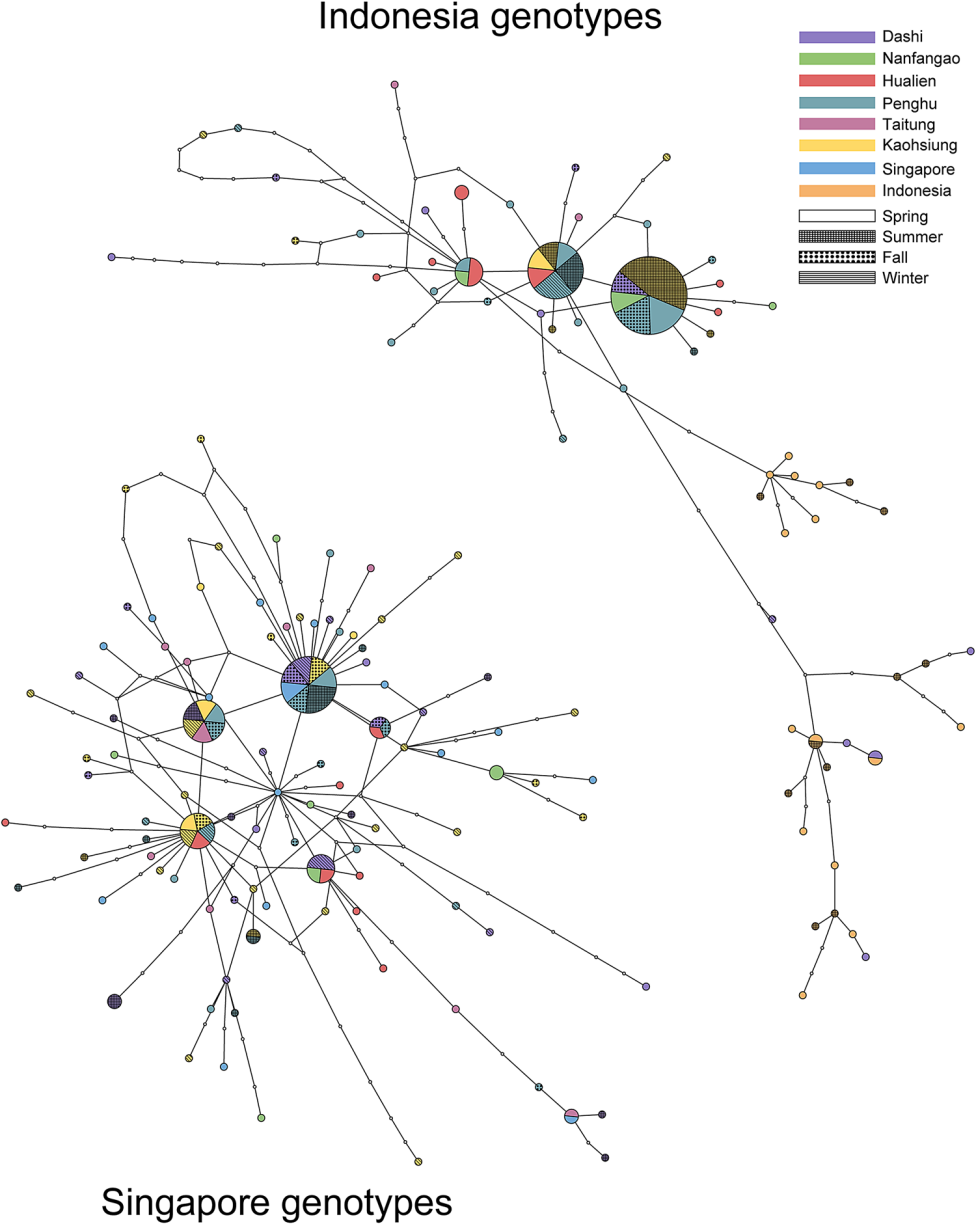
The TCS network. The TCS analysis of the genotypes shows two disconnected networks each corresponding to the Indonesia genotypes and Singapore genotypes. The diameters of the pie charts show the relative sample size of the haplotypes of the control region. All the samples of different collection sites are color-coded. The patterns in circles represent different seasons that samples been collected.

The mtDNA control region of samples collected from the offshore waters of Taiwan, Singapore, and Indonesia (Fig. 1) indicated the existence of a Singapore genotype and an Indonesia genotype (Fig. 3). The TCS networks revealed that the genotyped samples from Taiwan can be assigned to two disconnected networks, each of which respectively presents a genotype similar to those in the Singapore and Indonesia samples (Fig. 3). During the summer, 100% of the samples collected from the KC (east of Taiwan) were of the Indonesia genotype, whereas samples collected from the KBC (west of Taiwan) included both genotypes. During the spring, while the KC was weakening and the CCC was running south (Fig. 1b), there was a mix of the two genotypes in the waters off of both coasts; however, there was a higher proportion of the Singapore genotype. During the other seasons, there were fluctuations in the proportions of the two genotypes in all areas of Taiwan (Fig. 1c).

### Haplotype diversity (Hd) and nucleotide diversity (π) of subpopulations

Among the 202 M*. cordyla* samples collected from the eight locations in this study, we identified 150 haplotypes, corresponding to an average haplotype diversity of Hd = 0.991 and average nucleotide diversity of π = 0.035 (Supplementary Table S3). The nucleotide diversity (π) in populations collected from the offshore waters of Taiwan (π = 0.025–0.036) were higher than those from populations collected adjacent to Singapore (Malacca Strait) and Indonesia (Banda Sea) (0.006 and 0.008 respectively). We observed a similar profile in terms of θ levels (Supplementary Table S3).

Table 1 presents seasonal fluctuations in the nucleotide diversity of samples from the west of Taiwan (Kaohsiung): warm seasons while the SCSC was running to the north (π = 0.023–0.028) (Table 1; Fig. 1a) and cooler seasons while the CCC was running to the south (π = 0.018–0.019) (Table 1; Fig. 1b). Among the samples collected off the east coast near Dashi, the lowest nucleotide diversity (π = 0.007) was recorded during the summer, which coincided with an ocean current moving consistently in the same direction and was strongest during the summer. Among the samples collected from Penghu where the KBC weakens during the cool season (Fig. 1b), the nucleotide diversity (π = 0.033–0.041) was consistently high throughout the year and peaked in the winter (π = 0.041) when KBC and SCSC both in effect of the offshore water near Penghu (Table 1 and Supplementary Fig. S1).

**Table 1:** Haplotype and nucleotide diversities for *M. cordyla* in different seasons from June 2000 to May 2001.

	Spring	Summer	Fall	Winter
**Dashi**				
Replicate	10	9	8	10
Ηaplotype	10	8	8	9
*H*_*d*_	1.000	0.972	1.000	0.978
*π*	0.037	0.007	0.038	0.018
*θ*	25.10	6.99	23.91	21.92
**Penghu**				
Replicate	18	10	10	7
Ηaplotype	17	8	9	6
*H*_*d*_	0.993	0.956	0.978	0.952
*π*	0.033	0.033	0.037	0.041
*θ*	18.61	20.50	20.50	24.90
**Kaohsiung**				
Replicate	5	10	9	19
Ηaplotype	5	6	8	19
*H*_*d*_	1.000	0.778	0.972	1.000
*π*	0.028	0.023	0.019	0.018
*θ*	24.96	18.73	20.24	21.17

### Hypothesis testing of diversification periods

**Figure 4:**
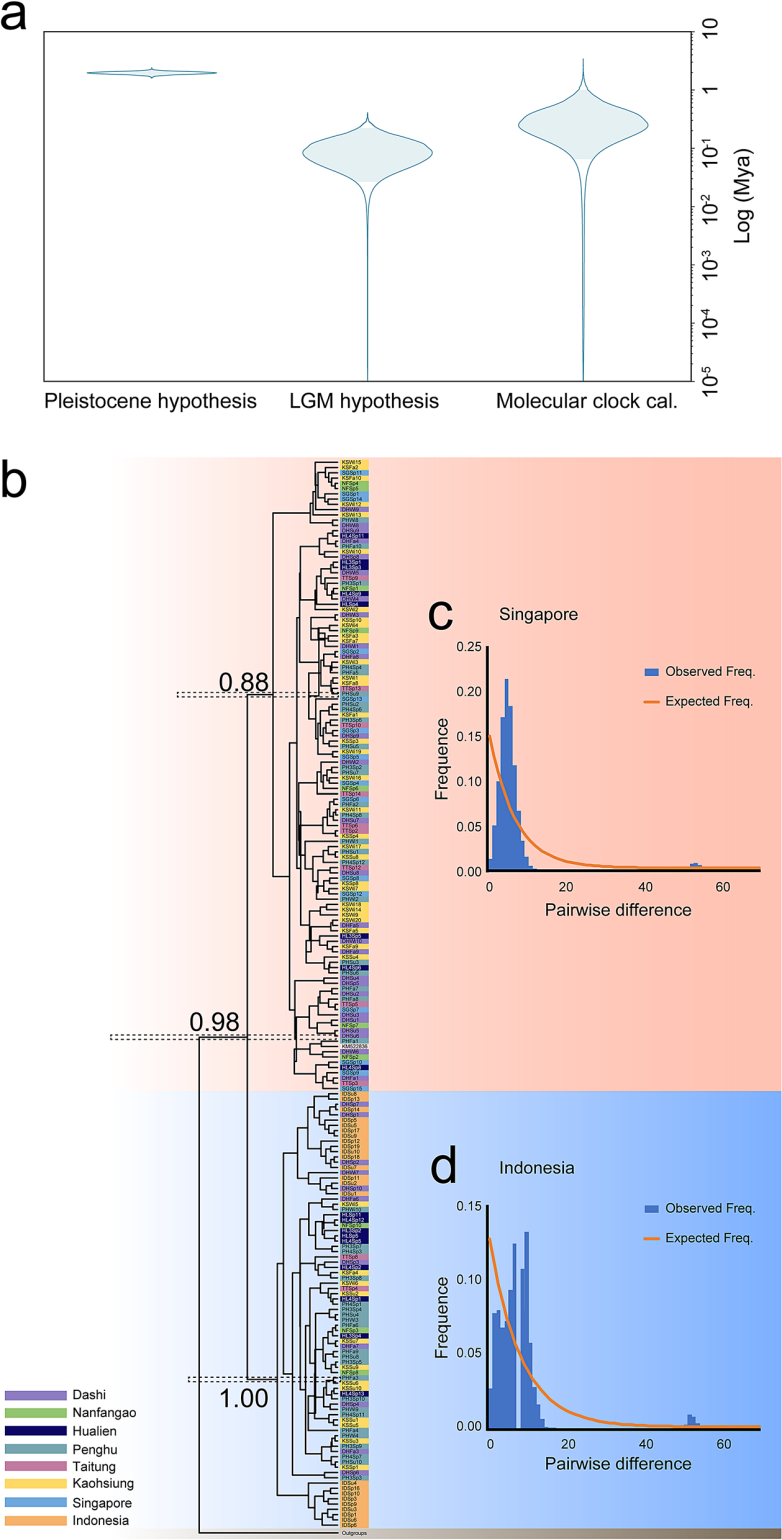
The results of time-calibrated phylogenetic analyses and the mismatch distribution of the Indonesia genotypes and Singapore genotypes. (**a**) The results of three divergence time hypotheses of the split between Indonesia genotypes and Singapore genotypes showing that the Pleistocene divergence hypothesis deviates from the other two hypotheses. (**b**) The BEAST maximum clade credibility tree calibrated using molecular clock showing two monophyletic clades corresponding to two genotypes. (**c**) and (**d**) are the results of the mismatch distributions of two clades, which both show signals of population demographic expansion, i.e., deviated from the curve under stable population size (showing in orange lines).

In our time-calibrated BEAST phylogenetic tree reconstruction, the samples clustered in the Singapore genotype and Indonesia genotype formed separated monophyletic clades (Fig. 4). A molecular clock configured to a rate of 3–10% genetic divergence in the control region per million years (My)^33,34^ revealed a split between the Singapore and Indonesia genotypes roughly 0.44 ± 0.287 My ago (Mya). The divergence date of *M. cordyla* populations calibrated using the time of the last glacial maximum (LGM)^35^ was 0.012 ± 0.055 Mya. The divergence date of *M. cordyla* populations calibrated using the Pleistocene event was 1.96 ± 0.010 Mya. BEAST analysis revealed that divergence estimates based on Pleistocene events were not realistic. Rather, it appears that the divergence date between the two genotypes was linked to a glacial episode (i.e., the LGM) (Fig. 4a).

Sequence mismatch analysis in the control regions revealed indications of previous expansion in both the Singapore and Indonesia clades. Samples from Singapore presented a unimodal mismatch distribution of pairwise nucleotide differences (Fig. 4c). The Indonesia clade presented a bimodal mismatch curve (Fig. 4d). The two populations carrying distinct mtDNA genotypes presented deviations from the mismatch distribution indicative of stable populations, which indicates that at least one of the populations recently underwent expansion (Fig. 4c,d).

## Discussion

A correlation was observed between seasonal ocean current cycles and the genetic diversity of *M. cordyla* (mtDNA genotypes) captured throughout the study period (2000–2001). While flowing toward the north, the KC carries a fish population from the Banda Sea near Indonesia. Depending on the strength of the SCSC, another population from the South China Sea near Singapore is carried north mainly toward the west of Taiwan (Figs. 1a, 3). The divergence date of the two mtDNA cohorts was estimated at 0.5 Mya, which may be associated with the glacial episodes before the LGM isolating the Banda Sea population from the South China Sea population. Our data obtained in 2000–2001 did not reveal any spawning samples in the offshore waters of Taiwan; however, 10 larvae were caught in 2.2–6.1 mm TL between June and August along the northernmost coast of Taiwan during 2008–2014^11^. This may explain why the two mtDNA cohorts are still genetically isolated, despite the fact that they coexist in the offshore waters of Taiwan. Sequence mismatch analysis revealed indications of population expansion in both mtDNA cohorts. Overall, our results revealed that time-series sampling in the same localities pertaining to GSI, genetic diversity, divergence time, and population size variations are crucial to the establishment of feasible conservation and fishery plans for the management of *M. cordyla* as well as other pelagic organisms. Our findings also provide important information related to the management of other pelagic species under the fluctuating KC, KBC, SCSC, and CCC currents. Despite the fact that these results are based on sampling from a single year, they clearly illustrate the importance of seasonal sampling in efforts to unravel the genetic diversity in pelagic ecosystems.

Numerous researchers have sought to explain genetic variability between populations of marine species at the local and regional scales. Most existing theories focus on the influence of ocean currents and major oceanic gyres limiting larval dispersal, whereas other theories focus on localized natural selection against immigrants^36^. Planes suggested that genetic restructuring may be associated with the vertical and/or horizontal movement of larval fish in the water column to exploit eddies and return to natal reefs^37^. Nonetheless, the role of ocean currents as an oceanographic barrier in the subdivision of marine organism populations has yet to be adequately elucidated. Note that theories based on ocean currents are difficult to test, due to the uncertain linkages among spawning area, seasonal migration patterns, and oceanographic phenomena. This study demonstrated that these questions can be addressed by sampling at particular locations over timespans sufficient to account for seasonal changes in ocean currents as long as information pertaining to spawning areas is available. If the genetic structure of a given species varies with the ocean currents, then it is reasonable to assume that seasonal fluctuations in flow fields would produce corresponding variations in the genetic structure at the intersection of ocean currents. Our genotyping of *M. cordyla* samples collected over a one-year period revealed that the species identified in any given location depend largely on the ocean currents at the time. Fishery plans will need to take these cyclic oceanographic factors into account.

Taiwan is an island affected by the KC, KBC, SCSC and CCC currents. The seasonal patterns in these ocean currents no doubt also affect the migration and commercial landings of other marine species (migratory and non-migratory)^29^. Pelagic organisms often vary in terms of genetic structure. For example, *Scomber australasicus* around Taiwan (Lee, unpublished data) present a homogeneous genetic structure. By contrast, *Decapterus macrosoma* exhibits significantly different genetic structures between populations in some adjacent regions^27^ (e.g., Sunda Strait vs. Java Sea) as well as notable genetic homogeneity over extensive regions from the South China Sea to Sulawesi Sea via the Java Sea and Makassar Strait. The geographical isolation of the *D. macrosoma* population in the Sunda Strait coincides with the presence of a sharp transition zone between the Indian Ocean and the Sunda Shelf. Researchers have also obtained evidence indicating that differences in the genetic structure of discrete populations may also be attributed to differences in salinity in different ocean currents^27^.

The *M. cordyla* in this study presented at least two well-structured genetic populations (Singapore and Indonesia), based on structures identified in the mtDNA control region. From the spawning areas near the equator, the Singapore population is carried northward by the SCSC whereas the Indonesian population moves with the KC^12^. The pronounced seasonal changes in the Kaohsiung and Dashi populations (affected by the KC) were not observed in the Penghu population. Nucleotide diversities in the Kaohsiung samples were higher in the spring (π = 0.028) and in the summer (π = 0.023) when the SCSC shifted northward, and subsequently decreased in fall (π = 0.019) and winter (π = 0.018) when the CCC reversed southwardly. We argued that the high nucleotide diversity pattern near Kaohsiung can be attributed to the multi-directions of the ocean current trajectories near Kaohsiung (Fig. S1) caused by the vortex of the KC and SCSC with the mixture of two genotypes. We also observed seasonal fluctuations in the nucleotide diversity of the Dashi sample; however, the lowest nucleotide diversity (winter, π = 0.018 and summer, π = 0.007) occurred when the direction of ocean currents was constant. We infer that the lowest nucleotide diversity in the summer could be attributed to the strengthen of KC during summer thus brings the Indonesian genotypes to Dashi offshores while SCSC has less effect thus no Singapore genotypes arriving to the northern Taiwan sampling site. Nucleotide diversity in the Penghu sample was high throughout the year (π = 0.033–0.041), which could be attributed to the vortex of KBC and CCC especially during the winter near Penghu. Overall, changes in the genetic structure of *M. cordyla* around Taiwan (within and among populations) were strongly influenced by KC and less so by the CCC.

Some widely dispersed marine organisms present a highly homogeneous genetic structure resulting from the gene flow among geographic regions^17–22^. The *M. cordyla* in the current study is also widely dispersed; however, there is clear genetic differentiation between populations. The genetic differentiation of other pelagic organisms in the West Pacific region have been attributed to glacial events^27,29–31^. Our divergence time estimates based on time-calibrated phylogenetic analysis indicate that the Singapore and Indonesia genotypes can probably be attributed to the glacial periods short before the LGM event associated with the formation of a land bridge separating the South China Sea and South Pacific Ocean. Subsequent interglacial periods no doubt expanded the range and the population size of both genetic cohorts, resulting in the distributions observed today. However, whether these two genetic cohorts have gone through speciation event, which is difficult to test using mitochondrial data that we currently have, need to be further tested using multiple nuclear makers or genomic data.

This population genetics study of *M. cordyla* in the offshore waters of Taiwan demonstrated the importance of time-series sampling of migratory pelagic fish, which are often assumed to be genetically homogeneous. Our results are not representative of long-term fluctuations in the genetic structure of *M. cordyla*; however, our results clearly indicate the need to invest in multi-year monitoring for similar pelagic marine organisms. A failure to account for genetic variations over extended timespans could greatly curtail the formulation of plans for the conservation of fish stocks. To do the long-term and large sample size monitoring, the application of the cost-effective genotyping methods, e.g., genomic methods, need to be considered. Our findings may be applicable to other pelagic fish species in these area as well as other oceanic organisms existing under similar oceanographic systems.

## Material and methods

### Specimen sampling

The *Megalaspis cordyla* samples around Taiwan were collected monthly during June 2000 to May 2001 from the offshores near the east of Taiwan, i.e., Dashi, where KC runs to the north year-round; from the offshores of Kaohsiung, where KBC reaches year-round; and from the offshores of Penghu, where KBC does not arrive in winter (based on the − 20 m data from Shipboard Acoustic Doppler Current Profiler, SADCP^38^, Fig. 1 and Supplementary Fig. S1). The samples from these three localities are grouped by seasons that correspond to the seasonal activities of ocean currents. In spring, i.e., March to May, SCSC is directed to the north, and the KBC on western Taiwan is strengthening. In summer, i.e., June to August, the SCSC continues to move northward, and the power of KBC on western Taiwan reaches the highest. In fall, i.e., September to November, the CCC commence changing its direction toward the south, and the KBC on western Taiwan become less prominent. In winter, i.e., December to February, the CCC continues toward the south, and the KBC on western Taiwan becomes weakest or stopped near Penghu (Fig. 1). We also sampled the offshores near Nanfangao, Hualien and Taitung in spring 2001 (Fig. 1c). Other than the samples collected around Taiwan, we also collected samples at the source populations from Indonesia (near the Banda Sea, 6.1333° S, 128.3167° E), and Singapore (near Malacca Strait, 4.6626° N, 99.5451° E).

### Gonadosomatic index (GSI)

The study was approved by the Institutional Animal Care and Utilization Committee of Academia Sinica, Taiwan. All methods were performed in accordance with the applicable guidelines for the care and use of animals. We measured the gonadosomatic index using the equation (gonad weight (g)/body weight (g)) × 100% of the collected samples in each month from the offshores of Taiwan. We analyzed the seasonal reproductive status using the mean GSI values of *M. cordyla* populations caught seasonally from the offshores of Taiwan. We used ANOVA (SAS 8.2, SAS Institute, USA) to compare seasonal changes of the mean GSI values. We did not calculate the GSI of the samples from Singapore and Indonesia because we only sampled the muscle tissues for molecular work. Furthermore, we also measured the body length of the collected samples from the offshores in Taiwan.

### Molecular work

We extracted the genomic DNA from muscle tissues using a Puregene DNA Isolation Kit (Gentra Systems, Inc., Minneapolis, MN, USA). The nucleic acid pellet was re-suspended in deionized water and frozen at − 20 °C until we conducted PCR. We conducted PCRs in a mixture of 50 μl reaction solution containing 5 μl of 10X reaction buffer (10 mM Tris HCl, pH9.0; 50 mM KCl; 15 mM MgCl_2_; 0.1% Triton X-100), 0.4 μM each of the primers, 0.2 μM each of dNTP, 50–100 ng crude DNA, and 1 unit Taq DNA polymerase (Takara kit, USA). We used two primers, namely P1 and PB, to amplify ~ 1.2 kb partial fragment of the control region, tRNA^phe^, and partial 12S RNA regimes. The following two internal primers for *M. cordyla* mitochondrial control region, corF and corB (corF: GAC TCT AAC TCC TGC CCC TAA; corB: GCA GGC ACA AAG GTT TGG TC), were designed for sequencing. The thermocycler conditions were initial denaturation for 2 min at 94 °C, 35 cycles of denaturation for 1 min, primer annealing for 1 min at 50 °C, and extension for 1.5 min at 72 °C, followed by final extension for 10 min at 72 °C. We conducted the sequencing using the automatic sequencer, Biosystems 377A (facility at Min-Hsin Corp., Taiwan). Chromatograms and contiguous alignments were edited using Seqman version 4.00 (DNAstar, Madison, WI, USA). All partial sequences of the control region were aligned using the Clustal W program^39^ in MegAlign (DNAstar), and then BioEdit version 4. 7. 8^40^.

### Molecular data analysis

We reconstructed the time-calibrated phylogenetic tree using BEAST v1.10.4^41^. The outgroup choice is based on the phylogenetics of Carangidae^42^. We used closely related species of *M. cordyla* from GenBank: *Alepes djedaba*, *A. kleinii*, *Atule mate*, *Uraspis helvola*, *U. secunda*, as the proximate outgroup^42^. We used the species from genus *Trachurus* (*T. declivis*, *T. japonicus*, *T. mediterraneus*, *T. novaezelandiae*, *T. picturatus*, and *T. trachurus*) as the distal outgroup. We set up three competing scenarios for time-calibration in the BEAST analyses, which are (1) the time-calibration based on the evolutionary rate, 3% to 10% per million years (My), of control region^33,34^; (2) the divergence of *M. cordyla* populations occurred in the last glacial maximum (LGM) in ~ 250,000 years ago^35^; (3) the divergence of *M. cordyla* populations occurred in the Pleistocene (~ 2.0 My ago, Mya). In the time-calibrated BEAST analyses, we applied an uncorrelated relaxed clock for independent branch rates, Yule model for speciation, and GTR + I + G model for nucleotide substation for all three BEAST analyses. We used a 100,000,000 chain length with 10,000 sampling frequency to collect at least 200 effective sample sizes (ESS). In the molecular clock analysis, we used ucld.mean = 0.065 and ucld.stdv = 0.01 in real space to constraint the molecular clock within 3–10% per My under the lognormal distribution. We dated the age of the ingroup (i.e., *M. cordyla* samples) in 0.025 My with arbitrary standard deviation 0.1 My for the LGM hypothesis. We dated the age of the ingroup in 2.0 My with arbitrary standard deviation 0.1 My for the Pleistocene hypothesis. We then compared the posterior distributions of the estimated divergence time of *M. cordyla* in the samples that we collected. Genealogical relationship network of haplotypes was prepared with TCS version 1.2^43^ and visualized in tcsBU^44^. Haplotype diversities and nucleotide diversities of the control region sequences from each subpopulation and season were calculated using DnaSp 6.12.03^45^. We also conducted the mismatch distribution analyses for testing the population size changes in the lineages that we detected in the phylogenetic analyses using DnaSp 6.12.03. All sequence data are deposited at GenBank (accessions MN849043-MN849132); the tree files are deposited at TreeBASE (submission ID 25561).

## Supplementary information

Supplementary Information.
