# Supplementary material for: Implication of single year seasonal sampling to genetic diversity fluctuation that coordinates with oceanographic dynamics in torpedo scads near Taiwan
Source: Sci Rep. 2020 Oct 8;10:16829. doi: 10.1038/s41598-020-74025-9 (PMC7544891; doi:10.1038/s41598-020-74025-9)

Submitted to: Scientific Reports

Article type: Original article.

Word count: 6563 words (abstract, text, references, and figure & table legends)

Abstract, 225 words; 4 figures; 1 table; 1 supporting information.

Title: Implication of single year seasonal sampling to genetic diversity fluctuation that coordinates with oceanographic dynamics in torpedo scads near Taiwan.

Yong-Chao Su[1], Shan-Hui Su[2], Han-Yun Li[1], Hurng-Yi Wang[3], and Sin-Che Lee[4*]

1. Department of Biomedical Science and Environmental Biology, Kaohsiung Medical University, Kaohsiung, 80708, Taiwan
2. Kaohsiung Municipal Zhongshan Elementary School, Kaohsiung, 80457, Taiwan
3. Institute of Clinical Medicine, National Taiwan University, Taipei, 10617, Taiwan
4. Institute of Cellular and Organismic Biology, Academia Sinica, Taipei, 11529, Taiwan

Table S1. The seasonal variation of gonadosomatic index (GSI) value and body length in the offshore of Kaohsiung, Dashi and Penghu

	Spring	Summer	Fall	Winter	p-value
**Dashi**					
GSI (%) – ♀	0.75 ±0.40[a]	1.55 ±0.31[b]	0.83 ±0.44[a]	0.78 ±0.37[a]	0.00012
Body length (cm) – ♀	31.73 ±0.78[a]	26.13 ±0.71[c]	33.27 ±2.67[ab]	34.93 ±3.49[a]	0.0001
N	13	5	20	33	
GSI (%) – ♂	0.39 ±0.16[a]	1.51 ±0.79[b]	0.65±0.71[a]	0.13 ±0.05[a]	0.0002
Body length (cm) – ♂	31.72 ±1.02[a]	26.90 ±1.27[b]	32.15 ±2.99[a]	33.93 ±3.22[a]	0.0006
N	7	4	23	17	
**Penghu**					
GSI (%) – ♀	0.95 ±0.70[a]	2.20 ±1.71[b]	1.21 ±1.22[a]	0.41 ±0.30[a]	0.0124
Body length (cm) – ♀	29.50 ±8.16	29.00 ±13.22	28.79 ±6.39	19.14 ±18.86	0.2088
N	34	9	24	6	
GSI (%) – ♂	1.44 ±1.22[b]	1.92 ±0.64[c]	1.05 ±0.67[b]	0.09 ±0.04[a]	0.00023
Body length (cm) – ♂	27.86 ±1.03[ab]	30.77 ±1.72[b]	28.53 ±0.96[a]	28.96 ±1.13[ab]	0.0015
N	43	6	26	6	
**Kaohsiung**					
GSI (%) – ♀	0.44 ±0.26[b]	5.41 ±3.30[c]	2.06 ±1.45[c]	0.39 ±0.26[a]	0.0001
Body length (cm) – ♀	25.05 ±3.72[bc]	25.07 ±0.92[c]	28.89 ±4.00[a]	26.66 ±4.00[b]	0.0001
N	43	27	40	70	
GSI (%) – ♂	0.49 ±0.58[a]	1.89 ±0.93[c]	1.40 ±1.35[b]	0.14 ±0.11[a]	0.0001
Body length (cm) – ♂	24.60 ±3.27[b]	24.42 ±1.19[b]	27.45 ±4.26[a]	27.05 ±3.73[a]	0.0001
N	42	38	77	42	

Table S2. The list of sample collection, haplotypes, accession numbers, and clades.

Haplotype name	Number of individual	Sample names	Accession Number	Clade
Cor_1	7	DHSp1, DHSp7, IDSp14, IDSp17, IDSu5, IDSu8, IDSu9	MN849043	Indonesia
Cor_2	1	DHSp10	MN849044	Indonesia
Cor_3	1	DHSp2	MN849045	Indonesia
Cor_4	1	DHSp3	MN849046	Indonesia
Cor_5	6	DHSp4, DHFa6, PH4Sp3, HL4Sp12, HLSp11, NFSp10	MN849047	Indonesia
Cor_6	1	DHSp5	MN849048	Singapore
Cor_7	1	DHSp6	MN849049	Indonesia
Cor_8	1	DHSp8	MN849050	Singapore
Cor_9	29	DHSp9, DHFa4, DHFa8, DHWi1, DHWi3, DHWi9, KSSp10, KSSp3, KSFa10, KSFa8, KSFa1, KSWi1, KSWi12, KSWi15, KSWi19, PH3Sp6, PH4Sp4, PHSu2, PHSu9, PHFa10, PHFa5, HL4Sp11, NFSp4, NFSp5, SGSp1, SGSp13, SGSp2, SGSp3, SGSp5	MN849051	Singapore
Cor_10	2	DHSu1, NFSp7	MN849052	Singapore
Cor_11	1	DHSu2	MN849053	Singapore
Cor_12	8	DHSu3, PHFa7, PHFa8, TTSp5, SGSp10, SGSp15, SGSp7, SGSp9	MN849054	Singapore
Cor_13	1	DHSu4	MN849055	Singapore
Cor_14	2	DHSu5, DHSu6	MN849056	Singapore
Cor_15	8	DHSu7, KSSp4, KSWi11, PH4Sp8, PHFa2, TTSp2, TTSp6, SGSp6	MN849057	Singapore
Cor_16	1	DHSu8	MN849058	Singapore
Cor_17	1	DHSu9	MN849059	Singapore
Cor_18	2	DHFa1, TTSp3	MN849060	Singapore
Cor_19	1	DHFa3	MN849061	Indonesia
Cor_20	1	DHFa5	MN849062	Singapore
Cor_21	27	DHFa7, KSSp1, KSSu1, KSSu10, KSSu2, KSSu3, KSSu6, KSSu7, KSSu9, PH3Sp10, PH3Sp4, PH3Sp5, PH3Sp9, PH4Sp7, PHSu10, PHSu4, PHFa3, PHFa4, PHFa6, PHFa9, PHWi3, PHWi4, HL4Sp1, HL4Sp13, NFSp3, NFSp8, TTSp4	MN849063	Indonesia
Cor_22	13	DHFa9, DHWi10, KSSp8, KSSu8, KSFa5, KSWi20, KSWi17, PHSu1, PHSu6, PHWi2, HL3Sp5, HL4Sp6, TTSp12	MN849064	Singapore
Cor_23	2	DHWi2, TTSp14	MN849065	Singapore
Cor_24	7	DHWi4, DHWi5, PH3Sp1, HL3Sp3, HLSp4, NFSp1, TTSp9	MN849066	Singapore
Cor_25	1	DHWi6	MN849067	Singapore
Cor_26	1	DHWi7	MN849068	Indonesia
Cor_27	1	DHWi8	MN849069	Singapore
Cor_28	1	KSSu4	MN849070	Singapore
Cor_29	1	KSSu5	MN849071	Indonesia
Cor_30	1	KSFa2	MN849072	Singapore
Cor_31	1	KSFa3	MN849073	Singapore
Cor_32	2	KSFa4, PH3Sp8	MN849074	Indonesia
Cor_33	1	KSFa7	MN849075	Singapore
Cor_34	1	KSFa9	MN849076	Singapore
Cor_35	1	KSWi10	MN849077	Singapore
Cor_36	1	KSWi2	MN849078	Singapore
Cor_37	1	KSWi3	MN849079	Singapore
Cor_38	1	KSWi4	MN849080	Singapore
Cor_39	2	KSWi5, PHWi10	MN849081	Indonesia
Cor_40	1	KSWi6	MN849082	Indonesia
Cor_41	1	KSWi7	MN849083	Singapore
Cor_42	1	KSWi9	MN849084	Singapore
Cor_43	1	KSWi13	MN849085	Singapore
Cor_44	1	KSWi14	MN849086	Singapore
Cor_45	1	KSWi16	MN849087	Singapore
Cor_46	1	KSWi18	MN849088	Singapore
Cor_47	1	PH3Sp2	MN849089	Singapore
Cor_48	1	PH3Sp3	MN849090	Indonesia
Cor_49	1	PH3Sp7	MN849091	Indonesia
Cor_50	1	PH4Sp1	MN849092	Indonesia
Cor_51	1	PH4Sp11	MN849093	Indonesia
Cor_52	1	PH4Sp12	MN849094	Singapore
Cor_53	1	PH4Sp6	MN849095	Singapore
Cor_54	1	PHSu3	MN849096	Singapore
Cor_55	1	PHSu5	MN849097	Singapore

Haplotype name	No. of ind.	Sample names	Accession Number	Clade
Cor_56	1	PHSu7	MN849098	Singapore
Cor_57	1	PHSu8	MN849099	Indonesia
Cor_58	1	PHFa1	MN849100	Singapore
Cor_59	1	PHWi1	MN849101	Singapore
Cor_60	1	PHWi8	MN849102	Singapore
Cor_61	1	PHWi9	MN849103	Indonesia
Cor_62	1	HL3Sp1	MN849104	Singapore
Cor_63	1	HL3Sp2	MN849105	Indonesia
Cor_64	1	HL3Sp4	MN849106	Indonesia
Cor_65	1	HL4Sp2	MN849107	Indonesia
Cor_66	2	HL4Sp5, HLSp5	MN849108	Indonesia
Cor_67	1	HL4Sp8	MN849109	Singapore
Cor_68	1	HL4Sp9	MN849110	Singapore
Cor_69	1	NFSp2	MN849111	Singapore
Cor_70	1	NFSp6	MN849112	Singapore
Cor_71	1	NFSp9	MN849113	Singapore
Cor_72	1	TTSp13	MN849114	Singapore
Cor_73	1	TTSp10	MN849115	Singapore
Cor_74	1	TTSp8	MN849116	Indonesia
Cor_75	1	SGSp11	MN849117	Singapore
Cor_76	1	SGSp12	MN849118	Singapore
Cor_77	1	SGSp14	MN849119	Singapore
Cor_78	1	SGSp4	MN849120	Singapore
Cor_79	1	SGSp8	MN849121	Singapore
Cor_80	5	IDSp1, IDSp10, IDSp6, IDSu4, IDSu6	MN849122	Indonesia
Cor_81	3	IDSp11, IDSu1, IDSu2	MN849123	Indonesia
Cor_82	3	IDSp12, IDSp18, IDSu7	MN849124	Indonesia
Cor_83	1	IDSp13	MN849125	Indonesia
Cor_84	1	IDSp16	MN849126	Indonesia
Cor_85	1	IDSp19	MN849127	Indonesia
Cor_86	1	IDSp3	MN849128	Indonesia
Cor_87	1	IDSp5	MN849129	Indonesia
Cor_88	1	IDSp9	MN849130	Indonesia
Cor_89	1	IDSu10	MN849131	Indonesia
Cor_90	1	IDSu3	MN849132	Indonesia

Outgroup species name	Accession Number
Megalaspis_cordyla	KM522836
Alepes_kleinii	KF728081
Alepes_kleinii	EU131014
Alepes_djedaba	EU131018
Atule_mate	KM522838
Uraspis_helvola	KM978993
Uraspis_secunda	KT819204
Trachurus_japonicus	HM212549
Trachurus_trachurus	EU246482
Trachurus_trachurus	EU246481
Trachurus_japonicus	HM212551
Trachurus_picturatus	EU246458
Trachurus_mediterraneus	EU246437
Trachurus_mediterraneus	EU246428
Trachurus_declivis	AY533476
Trachurus_novaezelandiae	AY533475
Trachurus_novaezelandiae	AY533475

Dashi, DH; Nanfangao, NF; Hualien, HL; Penghu, PH; Taitung, TT; Kaohsiung, KS; Singapore, SG; Indonesia, ID.

Spring, Sp; Summer, Su; Fall, Fa; Winter, Wi.

Table S3. Haplotype and nucleotide diversities for *M. cordyla* in different collection sites.

location	DH	NF	HL	PH	TT	KS	SG	ID	Total
Replicate	37	10	17	45	11	43	15	24	202
Haplotype	34	9	15	34	11	33	14	22	150
H_d	0.996	0.978	0.985	0.977	1.000	0.980	0.990	0.993	0.991
π	0.032	0.033	0.036	0.035	0.025	0.030	0.006	0.008	0.035
θ	22.04	21.56	18.04	18.98	21.85	18.95	7.38	8.03	24.99

Dashi, DH; Nanfangao, NF; Hualien, HL; Penghu, PH; Taitung, TT; Kaohsiung, KS; Singapore, SG; Indonesia, ID.

Figure S1. Drifting trajectory at 20m depth of different seasons around Taiwan offshores in 2000-2001. The data was measured by Shipboard Acoustic Doppler Current Profiler (SADCP) from Ocean Data Bank[38]. The lack of information on the eastern side of Taiwan results from the strong northeast seasonal wind during the cool seasons. Map created using QGIS 3.12.3[47] and was modified with Adobe Illustrator CC 2019[48] based on the geographical data from GADM database[46].

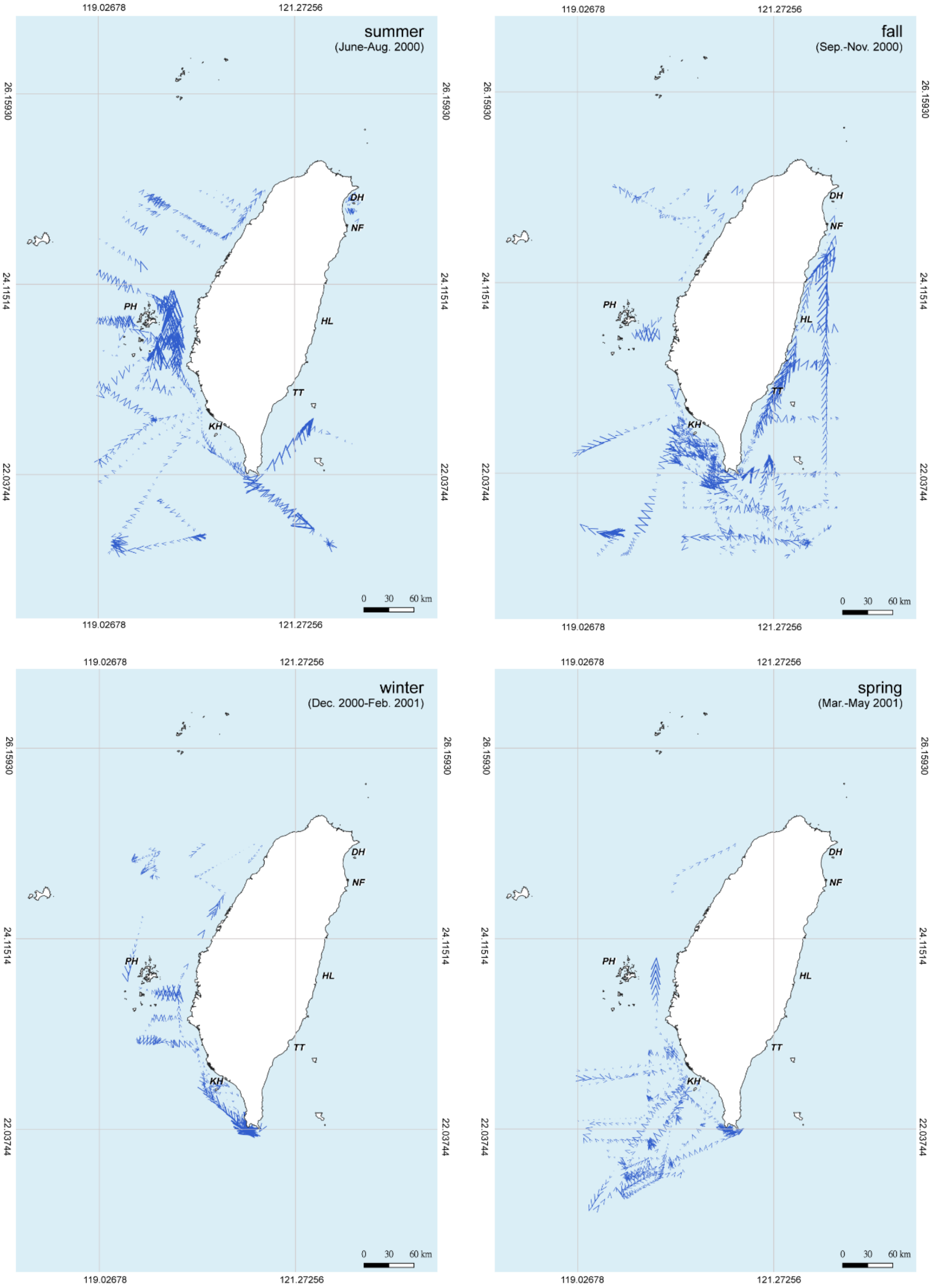

Supplement: Supplementary file 1 — Supplementary Information. [file 41598_2020_74025_MOESM1_ESM.pdf]
